# Carvedilol Reduces the Neuronal Apoptosis after Ischemic Stroke by Modulating Activator of Transcription 3 Expression in vitro

**DOI:** 10.1159/000527484

**Published:** 2022-10-11

**Authors:** Zhao Zheng, Fei Hou, Guodong He, Fengfeng Jiang, Xiang Bao, Minfeng Tong

**Affiliations:** ^a^Department of Neurosurgery, Jinhua Municipal Central Hospital, Jinhua, China; ^b^Pathological Diagnostic Centre, Jinhua People's Hospital, Jinhua, China

**Keywords:** Activator of transcription 3, Ischemic stroke, Cell viability, Carvedilol, Cell apoptosis

## Abstract

Cerebral ischemia is divided into local cerebral ischemia and diffuse cerebral ischemia. The etiology of localized cerebral ischemia includes middle cerebral artery embolism; stenosis, occlusion, or thrombosis of extracranial internal carotid artery or vertebral artery; and cerebral artery spasm. The causes of diffuse cerebral ischemia include cardiac arrest, hypotension, anemia, and hypoglycemia. However, the underlying mechanism is still unclear. In this study, we demonstrated that activator of transcription 3 (ATF3) is a hubgene in IS by bioinformatics analysis. The expression of ATF3 was increased in PC12 cells with oxygen-glucose deprivation/reoxygenation (OGD/R) treatment. ATF3 deficiency inhibited cell viability and induced cell apoptosis, whereas ATF3 overexpression showed the opposite role in cell viability and cell apoptosis. Moreover, Carvedilol as a compound targeting ATF3 also facilitated cell viability and reduced cell apoptosis. ATF3 deficiency retarded the increase in cell viability and inhibition of cell apoptosis in OGD/R-PC12 cells with Carvedilol treatment. Additionally, the decreased Bax and cleaved caspase-3 were released in OGD/R-PC12 cells with Carvedilol and siATF3 treatment, while Bcl-2 expression was inhibited in OGD/R-PC12 cells with Carvedilol and siATF3 treatment. In conclusion, Carvedilol may be a key compound targeting ATF3 in OGD/R-PC12 cells.

**Graphical Abstract:**

Carvedilol positively regulated cell viability and negatively regulated cell apoptosis in OGD/R-PC12 cells by inhibition of ATF3.

## Introduction

Cerebrovascular disease is the main killer threatening human health worldwide, and its morbidity and mortality rate remain high. It has become the number one cause of death for residents in our country and seriously threatens the health of the people. In China, more than 2 million people suffer every year. More than 1.5 million of them died from this disease. With the improvement of the economic level and the changes of lifestyle, the incidence of the disease is still increasing year by year. Cerebrovascular diseases are divided into hemorrhagic and ischemic cerebrovascular diseases according to its clinicopathological characteristics. Among them, the highest incidence is ischemic stroke (IS), mainly from cerebral atherosclerosis. Inflammation, recovery, and recanalization of the ischemic penumbra surrounding the infarcted tissue are the basis of modern treatment for IS [1, 2].

Activator of transcription 3 (ATF3) is a member of the ATF/CREB family of transcription factors. Experiments have shown that ATF3 plays a dual role in cell apoptosis and cell cycle regulation, thereby affecting cell proliferation and tumor occurrence. ATF3 is a transcription factor with a leucine zipper structure (bZIP), which activates or inhibits transcription by forming a dimer through bZIP. ATF3 has been described as a stress-induced and adaptive response gene [3]. Generally, there is no expression of ATF3 in healthy and intact neurons, but is expressed when axons are damaged [4, 5]. In the peripheral nervous system, the increase and retention of ATF3 expression are related to neuroprotection and regeneration [6]. In addition, studies have found that ATF3 can protect the function of the optic nerve after injury [7].

Carvedilol helps block the oxygenation of coronary blood vessels mediated by nitric oxide [8]. Liu and Diogo et al. [9] further verified the neuroprotective effects of Carvedilol's antioxidant properties [10]. Areti et al. [11] showed the tendency of Carvedilol to counteract the oxidative stress caused by oxidized platinum in neuronal cells. However, there are currently no preclinical data to evaluate these properties of dextrobenzene Carvedilol, which makes it suitable for the treatment of patients with stroke and nerve injury. Therefore, this study aimed to investigate the potential protective effect of Carvedilol in an in vitro model of stroke nerve injury.

## Materials and Methods

### Cell Culture and Construction of Oxygen-Glucose Deprivation/Reoxygenation Model

PC12 was purchased from the ATCC, and primary neuronal cells (Cat. No. 1520) were purchased from ScienCell, USA. The construction of oxygen-glucose deprivation/reoxygenation (OGD/R) model PC12 cell lines was carried out as previously described [12]. Briefly, primary neuronal cells and PC12 cells were washed 3 times with PBS and then incubated with glucose-free RPMI 1640 medium and placed in an anaerobic chamber for 6 h with OGD stimulation at 37°C under a 95% N_2_/5% CO_2_ atmosphere. Finally, cells were cultured under the condition of neural basal medium containing 2% B27 and 10% fetal bovine serum under standard cell culture conditions (37°C, 5% CO_2_, and 95% air) at different time points (0, 24, 48 h), respectively. Each experiment was repeated 3 times, and data are expressed as mean ± SEM. All cell lines were maintained in DMEM with 10% FBS and 100 U/mL penicillin/streptomycin, and cultured under the supplier's direction.

### Bioinformatics Analysis

GSE128623 dataset was analyzed by Short Time-series Expression Miner. Then, gene clusters were analyzed by KEGG-GO as previously depicted [13]. Protein-protein interaction networks were analyzed by online software STRING (https://string-db.org/).

### RT-qPCR

TRIzol reagent (Ambion, CA, USA) was used to isolate total RNA. RNA reverse transcription and RT-qPCR were performed as previously depicted [14]. cDNA was reverse transcribed from 1 μg of total RNA using a reverse transcription kit (Takara, Japan). β-actin was used as an internal reference. Amplification was performed according to the instructions of 2 × SYBR Green Master Mix (Promega, USA). PCR was performed with an initial denaturation at 95°C for 30 s, followed by denaturation at 95°C for 30 s and annealing/extension at 60°C for 30 s for 40 cycles. Relative gene expression levels were calculated using the 2-ΔΔCt method of qRT-PCR. The primers from NCBI were listed as follows:

- ATF3 forward: 5′-TGCTCAGAGAAGTCGGAAGAA-3′;
- ATF3 reverse: 5′-TGGCACAAAGTTCATAGGGCA-3′;
- GAPDH forward: 5′-AGGTCGGTGTGAACGGATTTG-3′;
- GAPDH reverse: 5′-GGGGTCGTTGATGGCAACA-3′.

### Western Blotting

Protein was extracted using RIPA lysis buffer (: 25 mM Tris•HCl pH 7.6, 150 mM NaCl, 1%NP-40, 1% sodium deoxycholate, 0.1% SDS) supplemented with protease inhibitor cocktail. Then, proteins were denaturized, electrophoresized, transferred, immunoblotted, and visualized with chemiluminescent ECL reagent as previously described [15].

### Cell Viability and Apoptosis

PC12 cells were treated with a different reoxygenation time, Carvedilol, the indicated plasmids, and then seeded in 96-well plates. Cell proliferation was tested using CCK8 reagent (Sigma, Germany) according to the manufacturer's directions. PC12 cells were treated as CCK8 solution, and then collected, washed, stained with propidium iodide or/and annexin V (Sigma, Germany). Flow cytometry was used to analyze cell apoptosis.

### Target Drug Prediction

The compound targeting of ATF3 was predicted by QuartataWeb (http://quartata.csb.pitt.edu/), and the structure was predicted by PubChem (https://pubchem.ncbi.nlm.nih.gov). Then, we used the online software SwissTargetPrediction (http://www.swisstargetprediction.ch/) to analyze the targets of Carvedilol.

### Statistical Analysis

Statistical analyses were performed with unpaired Student's *t* test for two group comparisons and one-way ANOVA for multigroup comparisons by using the SPSS 22.0 Statistical Software. Data were presented as mean ± SEM of at least three independent experiments. A *p* value of 0.05 or less was considered to be significant.

## Results

### STEM Clustering Analysis of Main Gene Expression Trend and Function Analysis before and after Cortical Stroke

**Fig. 1:**
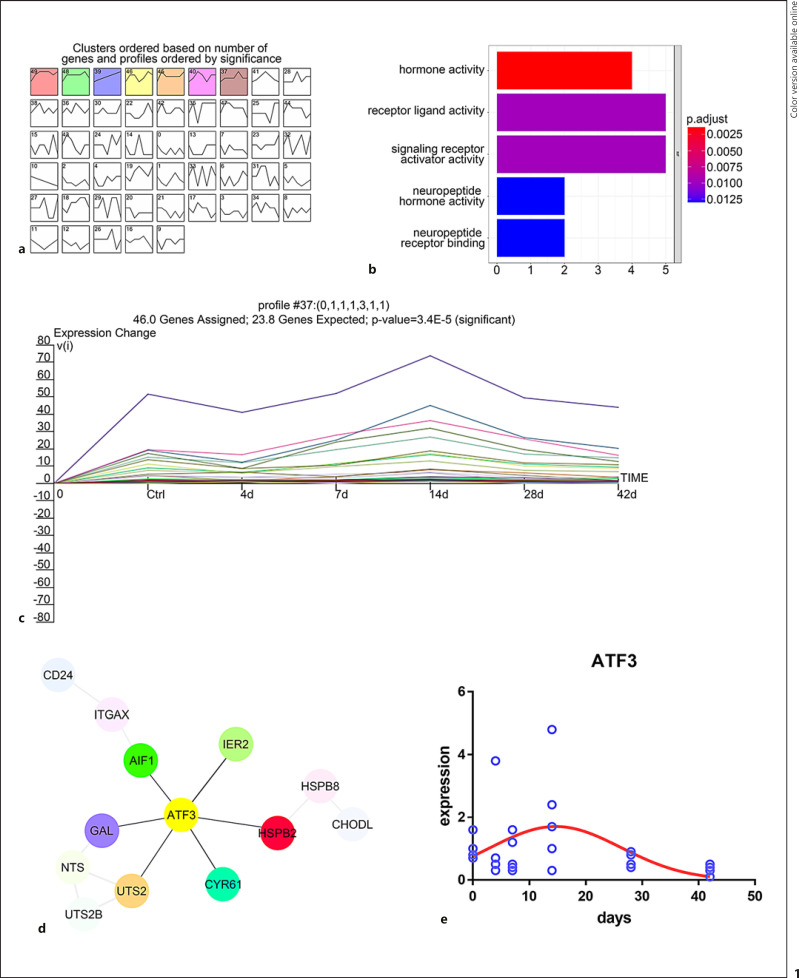
STEM clustering analysis of main gene expression and function analysis before and after cortical stroke. **a** Mainstream gene expression trends from GSE128623 were analyzed by STEM software. **b** The relationship between gene expression and time in profile 37 was analyzed by line chart. **c** KEGG-GO enrichment analysis of function genes in profile 37. **d** PPI network analysis of profile 37 by STRING. **e** The expression of hubgene in GSE 128623.

First, in order to clarify the underlined relationship between gene expression and time course, we used Short Time-series Expression Miner cluster to analyze the mainstream gene expression trends in GSE128623 (mice in ischemic injury model). As shown in Figure 1a, gene expression trends were significant in 7 profiles, including profile 37, 39, 40, 45, 46, 48, and 49. However, only profile 46 gene cluster expression showed an upward trend over time. Gene expression showed an upward trend in profile 37 (46 genes), indicating that the expression of genes in profile 22 was upregulated with the increase in the time of brain injury in mice (Fig. 1b). Therefore, we further used KEGG-GO to analyze the gene cluster in profile 37, and the most significant and abundant pathways of these genes related to neuromodulation were neuroligand-receptor interactions (Fig. 1c). Next, we used STRING to analyze the protein-protein interaction networks and searched the hubgene in profile 37. As shown in Figure 1d, ATF3 was a hubgene located at the core of the PPI network diagram. Moreover, the expression of ATF3 was increased with time (0–13 d), and the expression gradually decreased after 13 days (Fig. 1e).

### ATF3 Was Upregulated Expression in the OGD/R-PC12 Cells

**Fig. 2:**
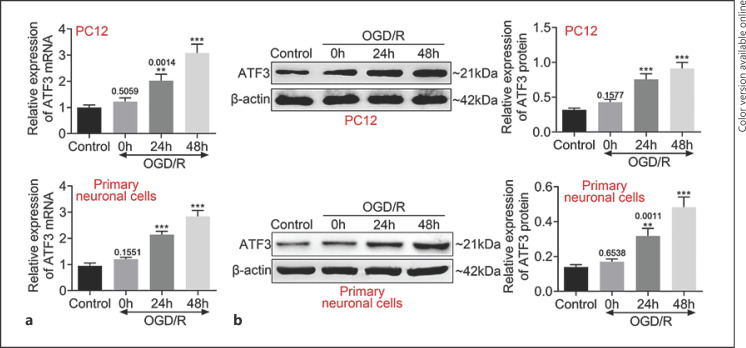
ATF3 was upregulated expression in the OGD model cells. **a** The expression of ATF3 mRNA in OGD/R-PC12 cells and OGD/primary neuronal cells was analyzed by real-time qPCR. **b** Western blotting was used to analyze ATF3 expression in OGD/R-PC12 cells and OGD/primary neuronal cells. Data are representative of three independent experiments (mean ± SD). **p < 0.01. ***p < 0.001. *p* values below 0.05 were considered significant. * compared to a control group.

Based on the core location of ATF3 in network of profile 22, we analyzed the expression of ATF3 in OGD/R model PC12 cells. As shown in Figure 2a, ATF3 mRNA expression was significantly upregulated in OGD/R model with reoxygenation treatment with 24 h and 48 h. Furthermore, the expression of ATF3 was increased over time. In addition, we also found the similar expression trends ATF3 at the protein level (Fig. 2b).

### ATF3 Promoted the Survival of OGD/R-PC12 Cells

**Fig. 3:**
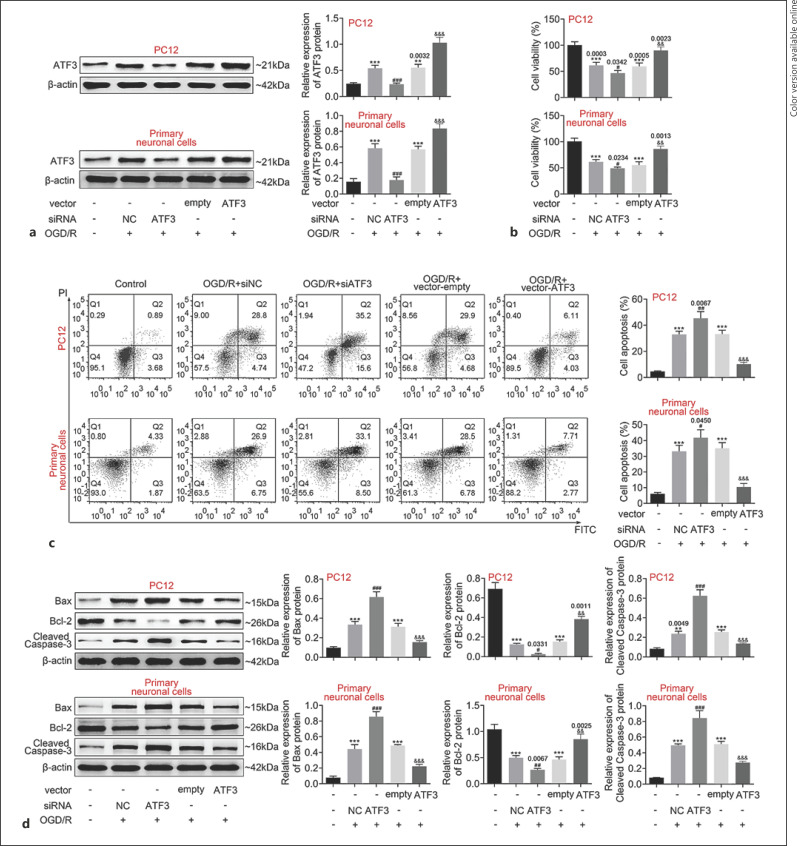
ATF3 promoted the survival of OGD model cells. **a** OGD/R-PC12 cells and OGD/primary neuronal cells (reoxygenation treatment 48 h) were transfected with the indicated plasmids; then, the expression of ATF3 was measured by Western blotting. **b** CCK8 assay was used to measure cell viability in OGD/R-PC12 cells and OGD/primary neuronal cells. **c** Cell apoptosis of OGD/R-PC12 cells and OGD/primary neuronal cells was measured by flow cytometry. **d** The expression of apoptosis-related proteins was measured by Western blotting. Data are representative of three independent experiments (mean ± SD). **p < 0.01. ***p < 0.001. ^#^*p* < 0.05. ^##^*p* < 0.01. ^###^*p* < 0.001. ^&&&^*p* < 0.001. * compared to a mock group, ^#^ compared with the siNC group. ^&^compared with empty group. A *p* value of 0.05 or less was considered as statistically significant.

Next, we want to clarify the function of ATF3 in cell level. First of all, siATF3 and ATF3 overexpression plasmids were transfected into OGD/R model PC12 cells and the transfection efficacies were confirmed by Western blotting (Fig. 3a). Then, cell viability and apoptosis were measured by CCK8 assay and flow cytometry analysis, respectively. As shown in Figure 3b, cell viability was reduced in OGD/R model PC12 cells with siATF3 treatment, and induced in OGD/R model PC12 cells with ATF3 overexpression. Downregulation of ATF3 facilitated cell apoptosis in OGD/R model PC12 cells, while overexpression of ATF3 inhibited cell apoptosis in OGD/R model PC12 cells (Fig. 3c). Furthermore, the expressions of Bax and cleaved caspase-3 were upregulated, and Bcl-2 was decreased in OGD/R model PC12 cells with siATF3 treatment. In contrast, overexpression of ATF3 promoted the expression of Bcl-2 and suppressed Bax and cleaved caspase-3 expression in OGD/R model PC12 cells (Fig. 3d). Collectively, our data suggested that ATF3 positively regulates cell survival in OGD/R model PC12 cells.

### Carvedilol Regulated the Survival and Apoptosis of OGD/R-PC12 Cells

**Fig. 4:**
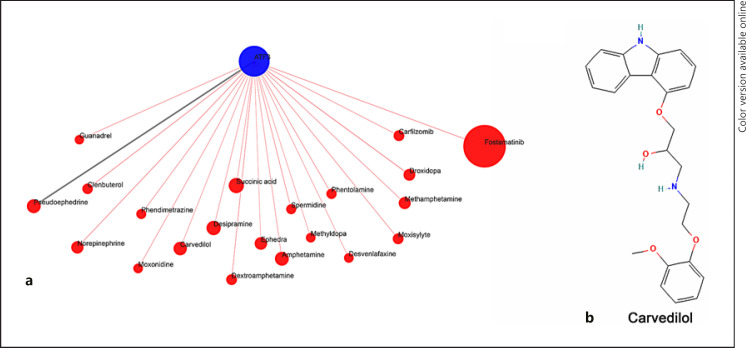
Compound-genomics target drug prediction for ATF3. **a** Compounds of targeted ATF3 were predicted by QuartataWeb (http://quartata.csb.pitt.edu/). **b** The structure of compounds was predicted by PubChem (https://pubchem.ncbi.nlm.nih.gov).

**Fig. 5:**
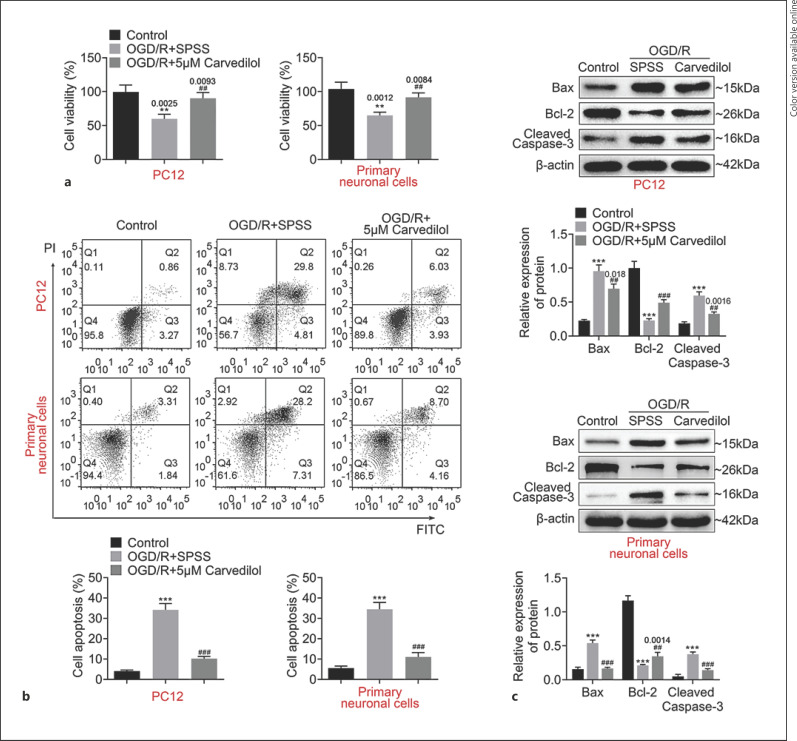
Carvedilol regulated the survival and apoptosis of OGD model cells. **a** Cell proliferation of OGD/R-PC12 cells and OGD/primary neuronal cells with Carvedilol treatment was measured by CCK8 assay. **b** Flow cytometry was used to measure cell apoptosis in Carvedilol-treated OGD/R-PC12 cells and OGD/primary neuronal cells. **c** Western blotting was used to measure Bax, Bcl-2, and cleaved caspase-3 expression in Carvedilol-treated OGD/R-PC12 cells and OGD/primary neuronal cells. Data are representative of three independent experiments (mean ± SD). **p < 0.01. ***p < 0.001. ^##^*p* < 0.01. ^###^*p* < 0.001. * compared to a mock group, ^#^ compared with the siNC group. ^&^compared with empty group. A *p* value of 0.05 or less was considered as statistically significant.

Based on the critical role of ATF3 in OGD/R model PC12 cells, we used QuartataWeb to predict the targeted compounds of ATF3. As shown in Figure 4a, there were 21 compounds targeting ATF3, including Carvedilol. Furthermore, we used PubChem to predict the structure of Carvedilol (Fig. 4b). Next, we measured the role of Carvedilol in cell viability and apoptosis. As shown in Figure 5a, the suppression of cell viability was retarded in OGD/R model PC12 cells with Carvedilol treatment and cell apoptosis was decreased in OGD/R model PC12 cells with Carvedilol treatment (Fig. 5b, c). Moreover, the expression of Bax and cleaved caspase-3 was both upregulated in OGD/R model PC12 cells and inhibited in Carvedilol-treated OGD/R-PC12 cells. The constraint of Bcl-2 expression was released by Carvedilol (Fig. 5d). Taken together, our data indicated that Carvedilol induces cell survival in OGD/R-PC12 cells.

### Carvedilol Regulated the Survival and Apoptosis of OGD/R-PC12 Cells by Targeting ATF3

**Fig. 6:**
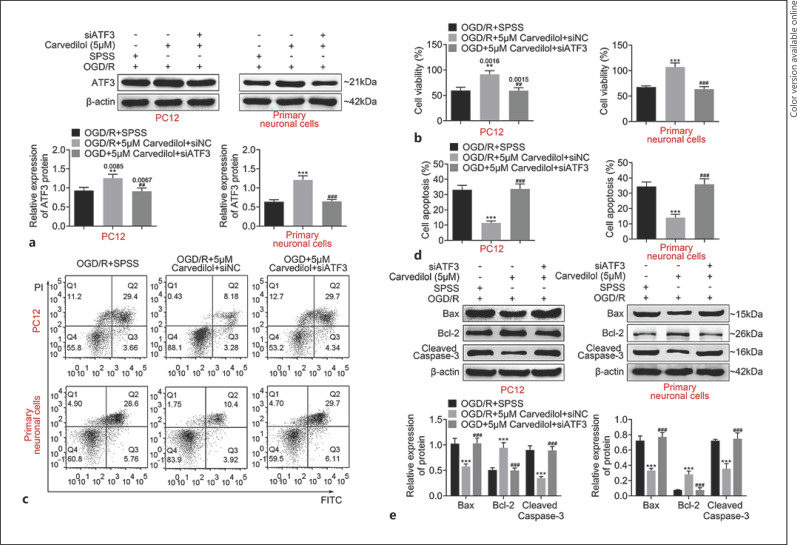
ATF3 deficiency reduced cell viability and induced cell apoptosis in OGD model cells with Carvedilol treatment. **a** Carvedilol-treated OGD/R-PC12 cells and OGD/primary neuronal cells were transfected with indicated plasmids, and ATF3 expression was measured by Western blotting. **b** Cell proliferation of ATF3 deficiency OGD/R-PC12 cells and OGD/primary neuronal cells with Carvedilol treatment was measured by CCK8 assay. **c, d** Flow cytometry was used to measure cell apoptosis in Carvedilol-treated ATF3 knockdown OGD/R-PC12 cells and OGD/primary neuronal cells. **e** Western blotting was used to measure Bax, Bcl-2, and cleaved caspase-3 expression in knockdown of ATF3 OGD/R-PC12 cells and OGD/primary neuronal cells with Carvedilol treatment. Data are representative of three independent experiments (mean ± SD). **p < 0.01. ***p < 0.001. ^##^*p* < 0.01. ^###^*p* < 0.001. *Compared to a mock group, ^#^Compared with the siNC group. ^&^compared with empty group. *p* values below 0.05 were considered as statistically significant.

For further exploring the function of ATF3 in OGD/R-PC12 cells with Carvedilol treatment, first, we measured the expression of ATF3 in OGD/R-PC12 cells with Carvedilol and siATF3 treatment. As shown in Figure 6a, ATF3 was highly expressed in OGD/R-PC12 cells while was downregulated in OGD/R-PC12 cells with Carvedilol and siATF3 treatment. Functionally, the increased cell viability was suppressed in OGD/R-PC12 cells with Carvedilol and siATF3 treatment (Fig. 6b). ATF3 deficiency antagonized the inhibition of cell apoptosis in OGD/R-PC12 cells with Carvedilol treatment (Fig. 6c). Furthermore, siATF3 treatment induced the expression of Bax and cleaved caspase-3 in OGD/R-PC12 cells with Carvedilol treatment, while the upregulation of Bcl-2 expression was inhibited in OGD/R-PC12 cells with Carvedilol and siATF3 treatment (Fig. 6d). Collectively, our data indicated that Carvedilol regulated the survival and apoptosis of OGD/R-PC12 cells through targeting ATF3.

## Discussion

IS is the main type of stroke, the main cause of which is lack of blood flow. Neuronal cell death caused by apoptosis is related to cerebrovascular stroke and various neurodegenerative diseases [16]. Drugs that maintain normal intracellular Ca^2+^ levels and inhibit cell oxidative stress may be effective in preventing abnormal neuronal apoptosis [17]. In this study, we constructed an OGD/R-PC12 cell model and identified hubgene − ATF3 in the GSE128623 dataset (mice in ischemic injury model). Through bioinformatics analysis, we next found that ATF3 was highly expressed in OGD/R-PC12 cells. ATF3 inhibits Bax and cleaved caspase-3, induced Bcl-2 expression, positively regulated cell viability, and negatively regulated apoptosis. Interestingly, ATF3 is a target of Carvedilol, which plays a similar role in regulating cell viability and apoptosis. Furthermore, ATF3 deficiency hinders the role of Carvedilol in regulating cell survival and apoptosis-related protein expression. ATF3 as an ATF/cyclic AMP response element binding series of proteins was expressed in a variety of cellular insults. ATF3 played multiple functions in the cell survival and cell death signaling cascade. Previous study reported that ATF3 is significantly overexpressed in brain ischemia from GSE22255 microarray dataset [18]. ATF3 exerted a critical role in regulation of caspase-dependent neuronal apoptosis signaling in focal cerebral ischemia-reperfusion injury [19]. In order to clarify the function of ATF3 in IS, we analyzed the expression of Bax, cleaved caspase-3, and Bcl-2 by Western blotting in the model of OGD/R-PC12 cells with ATF3 deficiency or overexpression. Bax is an evolutionarily conserved pro-apoptotic protein of the Bcl-2 protein family. The B-cell lymphoma 2 family members of apoptosis participated in cell survival and death, including Bax, Bad, and Bcl-2 [20]. The cleaved caspase-3 as a marker of apoptosis is an active form of caspase-3, which plays an important role in cell apoptosis cascades [21]. In addition, recent study demonstrated that extracellular vesicles derived from bone marrow mesenchymal stem cell origin carrying microRNA-221-3p protect against IS through ATF3, also suggesting a key role of ATF3 in IS [22]. Apart from this, ATF3 also regulated cell viability in OGD/R-PC12 cells. Next, we will elucidate the potential mechanisms by which ATF3 regulates cell survival and apoptosis.

Carvedilol contains two enantiomeric structures, both with antioxidant activity, and its unique new antioxidant properties can provide brain protection. Carvedilol not only provides safe and effective antihypertensive treatment, reduces the risk of stroke, but also better provides patients with additional benefits to prevent cerebral ischemia and stroke caused by the generation of oxygen-free radicals [23]. Previous research showed that Carvedilol can significantly reduce the infarct size and improve functional recovery after transient focal cerebral ischemia within a relatively large dose range [24]. Generally, Carvedilol as a β-adrenergic blocker exerted intrinsic antioxidant function and protected cardiac mitochondria from oxidative stress [9]. Although oxaliplatin-induced oxidative stress in nerve cells also exhibited free radical scavenging activity, Carvedilol was used in combination with oxaliplatin chemotherapy to prevent peripheral neuropathy [11]. Furthermore, Carvedilol reduced cell apoptosis signals by decreasing cytochrome C release and cleaved caspase-3 expression [10]. In our study, we demonstrated that Carvedilol inhibited cell apoptosis by reducing Bax and cleaved caspase-3 expression, and increasing Bcl-2 expression in OGD/R-PC12 cells. Moreover, knockdown of ATF3 released the inhibition of Bax and cleaved caspase-3, and retarded the upregulation of Bcl-2. However, the underlying functional regulatory mechanisms need further investigation.

In conclusion, we demonstrated that Carvedilol reduces apoptosis of OGD/R-PC12 cells by modulating ATF3 expression. ATF3 may be a key gene for Carvedilol in the treatment of ischemia stroke. However, how ATF3 modulates Carvedilol for IS remains to be further investigated.

## Statement of Ethics

The immortalized cell line PC12 and primary neuronal cells used in this study were obtained from ATCC and ScienCell (USA, Cat. No. 1520). Ethical approval for the use of these cells is not required in accordance with national guidelines.

## Conflict of Interest Statement

The authors state that there are no conflicts of interest to disclose.

## Funding Sources

This study is not supported by the funding.

## Author Contributions

Zhao Zheng and Fei Hou designed the study and supervised the data collection; Guodong He analyzed the data; Fengfeng Jiang, Xiang Bao, and Minfeng Tong interpreted the data, prepared the manuscript for publication, and reviewed the draft of the manuscript. All authors have read and approved the manuscript.

## Data Availability Statement

All data generated or analyzed during this study are included in this published article. Further inquiries can be directed to the corresponding author.
